# The Effect of Chrysin Nanocrystal on the Thyroid Gland of Rats Exposed to Chlorpyrifos

**DOI:** 10.2174/0118715303329277250120104421

**Published:** 2025-02-19

**Authors:** Tahereh Farkhondeh, Fatemeh Ahrari, Shahnaz Rajabi, Effat Alemzadeh, Behzad Mesbahzadeh, Maryam Rezaei, Sara Ziafati Majidi, Saeed Samarghandian

**Affiliations:** 1 Department of Toxicology and Pharmacology, School of Pharmacy, Birjand University of Medical Sciences, Birjand, Iran;; 2 Infectious Diseases Research Center, Birjand University of Medical Sciences, Birjand, Iran;; 3 Cardiovascular Diseases Research Center, Birjand University of Medical Sciences, Birjand, Iran;; 4 Department of Endocrinology, Faculty of Medicine, Birjand University of Medical Sciences, Birjand, Iran;; 5 Metabolic Syndrome Research Center, Mashhad University of Medical Sciences, Mashhad, Iran;; 6 Department of Medical Physioloy, School of Medicine, Health Ageing Research Centre, Neyshabur University of Medical Sciences, Neyshabur, Iran

**Keywords:** Chrysin, nanocrystal, chlorpyrifos, thyroid, oxidative stress, pest control

## Abstract

**Background:**

Chlorpyrifos (CPF) is an organophosphate insecticide that is mostly used in agriculture for pest control.

**Aim:**

This investigation aimed to evaluate the possible protective role of chrysin nanocrystals on thyroid gland hormones and histology in male rats after exposure to a high dose of chlorpyrifos.

**Methods:**

Rats were randomly divided into 6 groups (6 rats in each group): 1. healthy control group, 2. treated with chrysin nanocrystal (5 mg/kg), 3. treated with chrysin nanocrystal (10 mg/kg), 4. treated with chrysin nanocrystal (5 mg/kg) + chlorpyrifos, 5. treated with chrysin nanocrystal (10 mg/kg) + chlorpyrifos, and 6. treated with chlorpyrifos (30 mg/kg). After 15 days of intervention, rats were anesthetized, and blood samples were taken from the heart to measure thyroid hormones. Then, the thyroid gland was isolated and stored in 10% formalin for histopathological studies. Thyroid samples were also stored at -80°C for measuring oxidative stress parameters.

**Results:**

A significant reduction was observed in the serum concentrations of T3 and T4 in all treated groups compared with the control group (*p* < 0.01). In addition, hormone level examination revealed no statistically significant (*p* ˃ 0.05) changes in plasma TSH concentration in any of the groups. The treatment with CPF and chrysin nanocrystal did not affect the levels of oxidative biomarkers (MDA, GSH, and NO) in thyroid glands. Photomicrographs of a histological section of the thyroid gland showed vacuolar degenerated follicle epithelium and missing colloids in the histological section of the thyroid gland of all groups.

**Conclusion:**

Our findings demonstrated that the oral administration of chrysin nanocrystals could not inhibit the toxic effect of a high dose of CPF on the thyroid gland in the rats.

## 1 INTRODUCTION

Organophosphate pesticides (OPPs), such as chlorpyrifos (CPF), are effective against household pests [1, 2]. OPPs mostly cause acute toxicity by inhibiting acetylcholinesterase activity [3-5].

In addition, oxidative stress induced by OPPs has been implicated in their toxicity [6, 7]. Chronic exposure to CPF may be associated with serious damage to different organs [8]. CPF is also considered a major endocrine disruptor [9].

Many animal studies have reported that exposure to CPF during pregnancy or after birth induces neurobehavioral changes [10, 11]. Moreover, noncholinergic mechanisms have been shown to impair brain development [12, 13].

Some studies suggest that CPF may affect the homeostasis of the endocrine system, including the thyroid and other adrenal glands [2, 14].

Thyroid hormones are necessary for the normal physiological functions of several organs, including brain development [15], metabolism of protein, lipids, and carbohydrates, as well as the heart [16], and normal reproductive and gastrointestinal functions [17]. Notably, environmental exposure to CPF may affect thyroid function in humans [18]. Reduction in thyroxine (T4) levels and thyroid cellular changes have been found following CPF exposure [19]. Antioxidant supplementation could ameliorate the toxic effect of CPF on the thyroid glands.

Chrysin (5,7-dihydroxyflavone, CH) is a flavonoid that has been found in *Passiflora* species, honey, and propolis [20]. The pharmacological research on chrysin has indicated its anti-inflammatory, antioxidant, antidiabetic, anti-aging, and anti-cancer properties [20, 21]. The hydrophobic effect of chrysin leads to poor bioavailability and limits its clinical application. Nanocrystals represent a type of nanoformulation designed to enhance the solubility and bioavailability of poorly soluble compounds like chrysin. This study aimed to evaluate the protective effects of chrysin nanocrystals against chlorpyrifos-induced thyroid toxicity in male Wistar rats.

## 2 MATERIALS AND METHODS

### 2.1 Chemicals

CPF was obtained from Jiangsu Co., Ltd., China. Chrysin was purchased from Sigma Aldrich Co, USA. Oxidative stress kits (MDA, NO, and GSH) were purchased from Navand Salamat Co., Iran.

### 2.2 Preparation of Chrysin Nanocrystals

Chrysin nanocrystals (CHNC) were prepared according to the protocol of our previous study [22]. Chrysin nanocrystals were prepared using the solvent precipitation method. In this process, commercial chrysin was dissolved in acetone at predefined concentrations of 5 and 10 mg/ml. Then, a syringe was made and filled with the solution, and the syringe pump was tightened. Under magnetic stirring, the drug solution was injected rapidly (8 ml/min) into the anti-solvent solution (deionized water) at a ratio of 1/250 ml (300 to 1000 rpm). Filtered chrysin-nanocrystals were frizzed (-30ºC) and vacuum dried. The morphology of the chrysin-nanocrystals was examined using scanning electron microscopy (SEM) [22].

### 2.3 Experimental Design

This study was approved by the ethics committee of Birjand University of Medical Sciences (number: IR.BUMS.REC.1400.220). All animal research procedures followed were in accordance with the standards of Guide for the US National Research Council's and Guide for the Care and Use of Laboratory Animals.

In this study, 36 adult male Wistar rats (250 ± 23 g) were obtained from the “Experimental Medicine Center of Birjand University of Medical Sciences” and kept under standard conditions [22]. The animals were randomly selected and allocated into 6 groups (n = 6). Control (c), CPF CHNC 5, CHNC 10, CPF + CHNC 5, and CPF + CHNC 10 groups were administered olive oil (0.5 cc), CPF (30 mg/kg), CHNC (5 mg /kg), CHNC (10 mg /kg), CPF (30 mg/kg) with CHNC (5 mg/kg), and CPF (30 mg/kg) with CHNC (10 mg/kg) by gavage for 15 days, respectively.

At the end of the study, ketamine (60 mg/kg) and xylazine (10 mg/kg) were used for inducing anesthetized anesthesia in animals. Then, blood was obtained from the heart, and serum was separated for measuring thyroid hormones (T3, T4, and TSH). Furthermore, their thyroid tissues were removed for biochemical and histopathological evaluations.

### 2.4 Biochemical Assays

The levels of MDA, NO, and GSH in the thyroid samples were measured according to the protocol of standard kits (Navand Salamat). The concentrations of thyroid hormones (T3, T4, and TSH) in serum samples were measured by using the Pars Azmoon kit (DGKC).

### 2.5 Histopathological Assay

After sample collection, the thyroid tissue of rats was placed in formalin 10%. The samples were immersed in ethanol and then clarified with xylenol. Finally, the samples were placed in liquid paraffin at 60°C and prepared in sections of 4-5 μm. Hematoxylin-eosin (H & E) stain was used for our histology analysis. Three sections were prepared in each sample and evaluated by an experienced pathologist.

### 2.6 Statistical Analysis

Statistical calculations were performed using InStat 3.0 software. Data were shown as a mean standard error (SEM ± Mean). Using the Shapirville test, the hypothesis of normality of quantitative variables was examined [23]. If the assumption of population normality was confirmed and normality was established, statistical analysis of variance (ANOVA) and the Tukey tests were carried out to compare the data. A *p-*value of <0.05 was considered a criterion of significance.

## 3 RESULTS

### 3.1 SEM Analysis

The morphology of nanocrystals was described in our previous study [22].

### 3.2 Biochemical Findings

The serum concentrations of T3 and T4 significantly decreased in CPF, CHNC5, CHNC10, CPF + CHNC 5, and CPF + CHNC10 groups versus the control group (*p* < 0.01). No significant difference was observed in the serum concentrations of T3 and T4 between CPF and CHNC-5 and 10 treated groups exposed to CPF. A significant difference was not found between the serum TSH concentrations of CPF, CHNC5, CHNC10, CPF + CHNC 5, and CPF + CHNC10 groups versus the control group (*p* ˃ 0.05) (Table **1**). Furthermore, the serum concentrations of TSH in the CPF + CHNC 5 and CPF + CHNC10 did not change significantly from the CPF group.

CPF could not cause oxidative damage in the thyroid of male rats (Table **2**). CPF and CHNC 5 and10 mg/kg treatment did not affect the concentrations of GSH, MDA, and NO in thyroid glands in the CPF, CHNC5, CHNC10, CPF + CHNC 5, and CPF + CHNC10 groups versus the control group. The administration of CHNC 5 and 10 in CPF exposed group could not affect the concentrations of oxidative stress parameters versus the control group.

### 3.3 Histopathological Findings

**Fig. (1):**
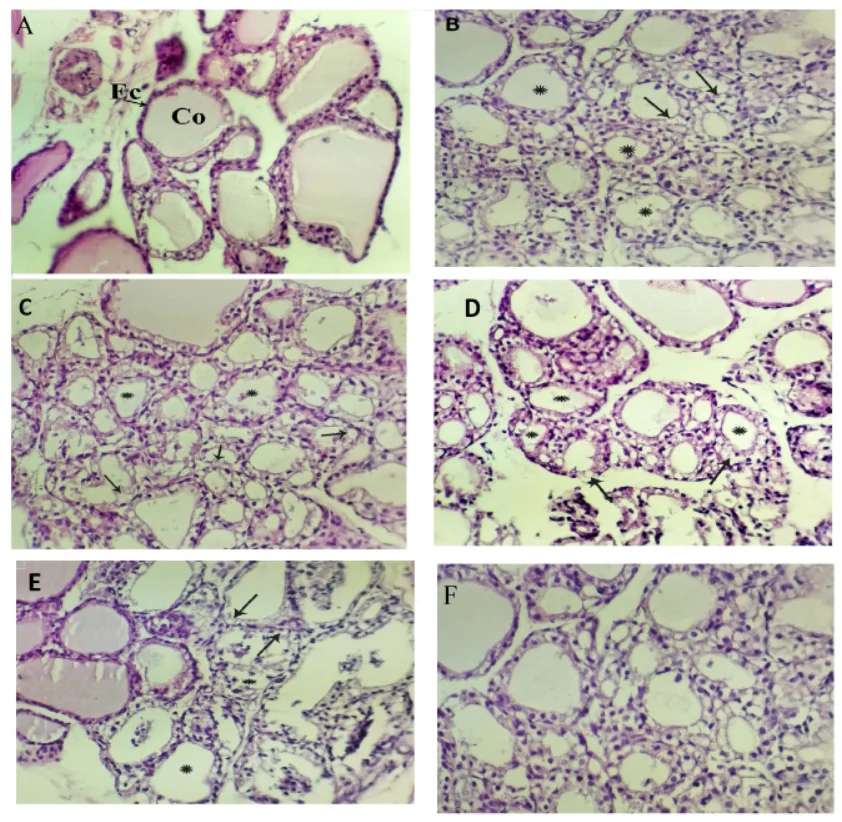
Photomicrographs of hematoxylin and eosin (H&E X 400)-stained sections of the thyroid of control rats (**1A**), CPF (**1B**), NCH5 (**1C**), NCH10 (**1D**), CPF + NCH5 (**1E**), CPF + NCH10 (**1F**) groups.

A healthy and normal histology was found in the control group (Fig. **1A**). Fig. (**1B**) shows vacuolar degenerated follicle epithelium and missing colloid in the histological section of the thyroid gland of the CPF group. Photomicrographs of the histological section of the thyroid gland of CHNC5, CHNC10, CPF + CHNC5, and CPF + CHNC10 showed vacuolar degenerated follicle epithelium and missing colloid (Figs. **1C**-**1F**).

## 4 DISCUSSION

Thyroid hormones are necessary for preserving normal physiological conditions in humans and animals [23]. Environmental pollutants, such as CPF, are able to disrupt thyroid function [19]. The objective of this study was to investigate the effects of chrysin nanocrystals on thyroid function in male Wistar rats exposed to high doses of CPF. In the current research, the high dose of CPF administration to the male rats reduced T3 and T4 concentrations, accompanied by no change in TSH concentration. The reduced serum T3 levels observed in the CPF group may be attributed to the low T4 levels recorded in this group rather than a decrease in hormone production, as T4 is converted to T3 to perform its biological functions [24]. Experimental studies have demonstrated that CPF also decreases serum thyroid hormone concentrations, particularly T4 [1]. The reduction effect of CPF on the serum T4 level may be caused by its direct toxic effect on the thyroid gland function and structure [25]. CPF has been found to induce oxidative damage and histopathological changes in the thyroid gland. In this context, El-Sheikh and Ibrahim [1] indicated that chronic administration of CPF caused changes in histology, including a decrease in vacuolated colloids, vacuolated follicular cells, and the size of follicles. Moreover, exfoliation of the follicular epithelial cells in the thyroid resulted in thyroid dysfunction. ROS generation and lipid peroxidation were caused by CPF, resulting in thyroid dysfunction [26]. Our findings demonstrated that CPF induced significant histopathological alterations in the thyroid gland, including follicular epithelial degeneration and colloid depletion. However, no changes were detected in the levels of oxidative stress parameters in the thyroid glands of animals exposed to a high dose of CPF for 15 days.

The mechanisms underlying CPF changes in thyroid hormones have not been fully understood. Previous studies reported that iodine-binding proteins may be reduced following insecticide exposure [27]. In addition, multiple studies have demonstrated that exposure to pesticides leads to oxidative stress [26, 28-30]. The controversy between our findings and previous reports may be mostly related to the duration of exposure. In our study, CPF could likely affect iodine-binding proteins at thyroid epithelial cells and reduce thyroperoxidase and deiodinase enzyme activities, consequently altering the histopathology of the thyroid gland and decreasing T4 production during 2 weeks [27].

We also found that the serum levels of T3 decreased in CPF, which was likely related to the low T4 level in this group. It might also be caused due to a decrease in the 5-deiodinase synthesis induced by CPF, resulting in a decrease in the conversion of T4 to T3.

Reduced serum levels of T4 and T3 induce TSH release *via* the pituitary-thyroid feedback mechanism. TSH has a significant effect on thyroid gland structure and function. It can elevate the size and vascularity of the gland. In addition, it increases iodine uptake and clearance from the plasma, iodotyrosine and iodothyronine formation, thyroglobulin proteolysis, and T4 and T3 secretion from the thyroid gland [31]. However, it was found that reduced levels of serum T3 and T4 could not increase TSH levels.

The TSH level was slightly reduced in the CPF group compared to the control group. However, this reduction was not statistically significant (*p* > 0.05), despite the low T3 and T4 levels due to a direct toxic impact of CPF on the nervous system, which was accompanied by acetylcholine accumulation and persistent post-synaptic cholinergic receptors activation [32]. Cholinergic activation induced somatostatin release, resulted in the inhibition of thyrotropin-releasing hormone (TRH) secretion and consequently suppressed the serum TSH levels [33].

Previous reports suggested that antioxidant supplementation effectively attenuated thyroid dysfunction by modulating oxidative stress. For this reason, we were interested in studying the effects of chrysin on thyroid function. Chrysin, a flavone nutraceutical with a wide range of beneficial effects, has critically low bioavailability on account of its poor aqueous solubility and, consequently, poor absorption from the gastrointestinal tract. It was found that the nanocrystal form of chrysin improved its antioxidant and anti-inflammatory activities [34]. Thus, we studied the effect of the nanocrystal form of chrysin on thyroid function.

Our findings revealed that chrysin nanocrystals did not inhibit the disruption of the thyroid hormones and histopathological damage elicited by the co-administration of CPF to the rats. In addition, we observed that chrysin nanocrystals decreased the T3 and T4 levels and also induced histopathological damage in animals that only received this agent at doses of 5 and 10 mg/kg. Experimental studies have indicated that flavonoids could inhibit the activity of the thyroperoxidase enzyme, leading to a decrease in thyroid hormone levels and histopathology alteration of the thyroid gland. Flavonoids could also change the thyroid hormone's availability to target tissues by inhibiting the activity of deiodinase or displacing T4 from transthyretin. Altogether, this study did not indicate the efficacy of the nanocrystal structure of chrysin against thyroid dysfunction. 

Molecular analyses are time-consuming and expensive, limiting the number of chemicals that can be tested and the ability to conduct molecular signaling tests to explore mechanisms.

## CONCLUSION

In summary, from the foregoing results, exposure to a high dose of CPF for 15 days caused thyroid toxic effects and resulted in a reduction in thyroid hormone levels in adult male rats. The co-administration of chrysin nanocrystal with CPF could not improve the thyroid structural state and the hormonal level. In addition, only administration of chrysin nanocrystal at doses of 5 and 10 mg/kg induced toxic effects on thyroid glands. Thus, chrysin nanocrystals have been indicated to interfere with thyroid hormone synthesis in *in vivo* models. It was not recommended to use chrysin nanocrystal supplementation as an antioxidant for overcoming the toxic effects of CPF. The lack of significant evidence for oxidative stress in this study may be attributed to the limited number of oxidative stress assays conducted. Specifically, only a few key markers of oxidative damage, namely MDA, GSH, and NO, were assessed in the thyroid tissue. Future studies should consider a broader range of oxidative stress markers and more comprehensive testing methods to provide a clearer understanding of the potential role of oxidative stress in CPF-induced thyroid dysfunction.

## AUTHORS’ CONTRIBUTIONS

T.F., F.A., Sh.R., E.A., B.M., M.R., S.Z., and S.S. took part in performing the experiments and implementation, as well as the data analysis and manuscript writing. T.F. and S.S. proposed the research design and critically revised the manuscript. All authors approved the final draft of the manuscript.

## Figures and Tables

**Table 1:** Comparison of thyroid hormones in experimental groups.

**Parameters**	**Groups**						
	**C**	**CPF**		**CHNC 5**	**CHNC 10**	**CPF+ CHNC 5**	**CPF+ CHNC 10**
T3	85.63 ± 2.46	68.30 ± 1.09 **		73.02 ± 2.05 *	71.39 ± 3.49 **	69.68 ± 3.19 **	70.60 ± 3.07 **
T4	17.51 ± 0.38	12.13 ± 1.38 **		13.50 ± 0.44 *	13.28 ± 0.80 *	12.81 ± 0.96 **	12.68 ± 0.84 **
TSH	0.015± 0.004	0.013 ± 0.003		0.016 ± 0.004	0.015± 0.003	0.005 ± 0.008	0.011 ± 0.002

**Note:** Data are shown as means ± SEM for each group (n = 6). C: control, CPF: chlorpyrifos, CHNC 5: chrysin nanocrystals (5 mg/kg), CHNC 10: chrysin nanocrystals (10 mg/kg), CPF+ CHNC 5:chlorpyrifos + chrysin nanocrystals (5 mg/kg), CPF+ CHNC 10: chlorpyrifos + chrysin nanocrystals (10 mg/kg). A significant difference between the data of the C group *vs.* other groups: *; *p* < 0.05, **; *p* < 0.01.

**Table 2:** Comparison of oxidative stress indices in experimental groups.

**Parameters**	**Groups**						
	**C**	**CPF**		**CHNC5**	**CHNC 10**	**CPF+ CHNC 5**	**CPF+ CHNC 10**
GSH (µM)	7.77 ± 0.19	7.78 ± 0.45		9.62 ± 0.48	8.67 ± 0.54	7.37 ± 0.77	8.53 ± 1.10
MDA (nM/mg tissue)	4.31 ± 0.22	6.42 ± 1.14		4.33 ± 0.38	4.70 ± 0.36	6.37 ± 1.13	6.45 ± 1.01
NO (µM)	10.37 ± 0.87	10.41 ± 0.61		10.27 ± 0.24	9.98 ± 0.11	11.07 ± 0.51	10.60 ± 0.56

**Note:** Data are shown as means ± SEM for each group (n = 6). C: control, CPF: chlorpyrifos, CHNC 5: chrysin nanocrystals (5 mg/kg), CHNC 10: chrysin nanocrystals (10 mg/kg), CPF+ CHNC 5:chlorpyrifos + chrysin nanocrystals (5 mg/kg), CPF+ CHNC 10:chlorpyrifos + chrysin nanocrystals (10 mg/kg).

## Data Availability

The data that support the findings of this study are available from the corresponding author, TF, upon reasonable request.

## LIST OF ABBREVIATIONS

CPF: Chlorpyrifos
OPPs: Organophosphate Pesticides
